# Protein crystallization: Eluding the bottleneck of X-ray crystallography

**DOI:** 10.3934/biophy.2017.4.557

**Published:** 2017-09-26

**Authors:** Joshua Holcomb, Nicholas Spellmon, Yingxue Zhang, Maysaa Doughan, Chunying Li, Zhe Yang

**Affiliations:** 1 Department of Microbiology, Immunology, and Biochemistry, Wayne State University School of Medicine, Detroit, MI, USA; 2 Center for Molecular and Translational Medicine, Georgia State University, Atlanta, GA, USA

**Keywords:** protein crystallization, X-ray crystallography, carrier mediated crystallization, PDZ scaffold mediated crystallization

## Abstract

To date, X-ray crystallography remains the gold standard for the determination of macromolecular structure and protein substrate interactions. However, the unpredictability of obtaining a protein crystal remains the limiting factor and continues to be the bottleneck in determining protein structures. A vast amount of research has been conducted in order to circumvent this issue with limited success. No single method has proven to guarantee the crystallization of all proteins. However, techniques using antibody fragments, lipids, carrier proteins, and even mutagenesis of crystal contacts have been implemented to increase the odds of obtaining a crystal with adequate diffraction. In addition, we review a new technique using the scaffolding ability of PDZ domains to facilitate nucleation and crystal lattice formation. Although in its infancy, such technology may be a valuable asset and another method in the crystallography toolbox to further the chances of crystallizing problematic proteins.

## 1. A Brief History

Protein crystallization was observed more than 170 years ago by Friedrich Ludwig Hünefeld with the unintended crystallization of hemoglobin from earth worm blood. This accidental finding was described in his book *Der Chemismus in der thierischen Organisation* (Chemical Properties in the Animal Organization) in 1840 [1, 2, 3]. However, it was not until the late 19^th^ century that scientists began to replicate the crystallization of proteins. Early protein crystallization attempts were used for purification of proteins. Scientists such as Funke in 1851 purified hemoglobin from red blood cells by dilution of red blood cells with solvents followed by slow evaporation to produce hemoglobin crystals [2, 3, 4]. Sequentially, botanists such as Ritthausen and Osborn implemented similar techniques in the 1880s through the 1890s to purify a series of plant seed proteins [2–6]. What was not realized at the time is that this accidental discovery would lend far more than the ability to isolate proteins from a sample but would become the foundation for the elucidation of high-resolution protein structure.

The investigation of molecular crystal structure dates as far back as 1611 when Johannes Kepler hypothesized the hexagonal crystal packing of snow in his work *Strena seu de Nive Sexangula* (A New Year’s Gift of Hexagonal Snow) [7]. However, it was not until the X-ray was discovered by Wilhelm Röntgen in 1895 that would make it possible to validate any proposed crystal models. In 1912, Max von Laue discovered the diffraction of X-rays by crystals. During the period of 1912–1913 William Laurence Bragg developed Braggs Law which describes the angles for coherent and incoherent scattering from a crystal lattice [8]. It was soon after that Bragg reported the first X-ray crystal structure of sodium chloride.

With X-ray diffraction in its infancy, the initial pioneers of protein crystallography focused on highly abundant proteins that could be produced and purified easily. The first protein structure to be solved was that of myoglobin from the sperm whale in 1958 followed by hemoglobin in 1960 and lysozyme from chicken egg whites in 1965 [9, 10, 11]. However, as the field progressed, scientists began to direct their efforts to objective-oriented projects involving proteins with different molecular weights and from different sources. It was then realized that the bottleneck of protein structure determination is the production of protein crystals suitable for X-ray diffraction.

## 2. The Premise of Protein Crystallization

**Figure 1:**
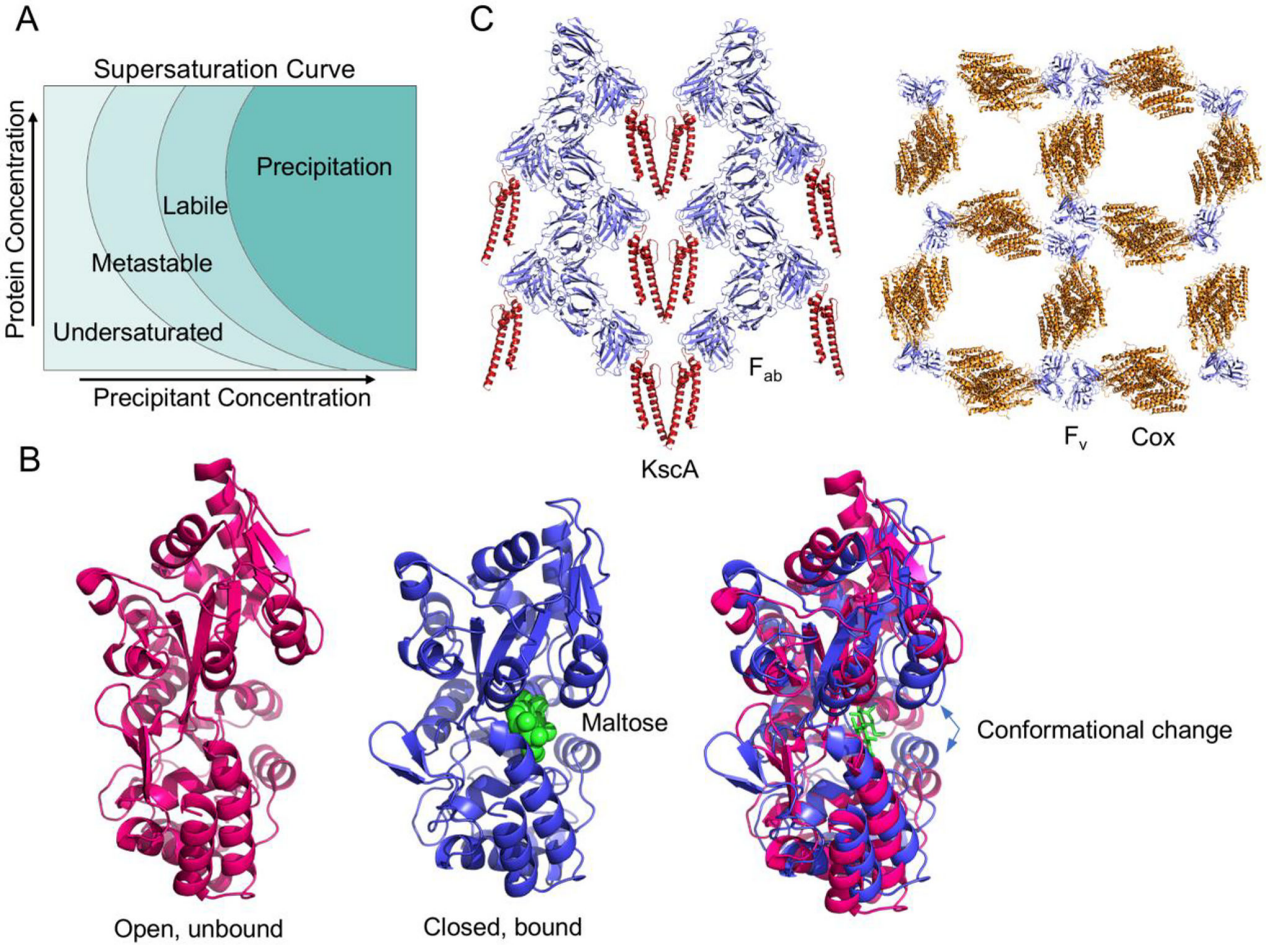
Carrier mediated protein crystallization. (A) The phase diagram for protein crystallization. (B) Conformational change of MBP upon binding of maltose. Unbound (left), bound (middle), and superposition of the unbound and bound forms (right). (C) Crystal lattice formation as mediated by antibody fragments. Left, the crystal lattice of KscA K^+^ channel mediated by an F_ab_ fragment (PDB code 1K4C); right, the crystal lattice of COX mediated by a recombinant F_v_ fragment (PDB code 1QLE).

Protein crystallization today is achieved by the same basic principle as was discovered over 170 years ago. Supersaturation of a protein in solution is the basis behind the crystallization. At the supersaturated state, the amount of proteins in solution exceeds their solubility limit. Under this non-equilibrium state, the proteins are being pushed out of the solution undergoing a first ordered phase transition known as nucleation. Supersaturation of a protein in solution can be achieved by several different methods. Usually, a chemical known as precipitant is used to reduce protein solubility and create the supersaturation state. The phase diagram (Figure 1A) demonstrates the dependence of increasing protein and precipitant concentration on the saturated state. At both low protein concentration and precipitant concentration, the protein remains in the stable, undersaturated state. As either protein or precipitant concentration is increased in solution, the protein can undergo a transition to either the metastable, labile, or precipitation phase [2, 3, 12]. In the metastable phase, nuclei may form, which are stable compared to the parent liquid phase and metastable compared to the crystalline phase of the protein [13]. The labile phase is where both nucleation and crystal growth may occur [14]. The precipitation phase is where the highest degree of supersaturation exists, in which ordered nucleation does not occur and there is no crystal growth. Thus, crystallization is dependent on the magnitude and rate at which supersaturation is achieved.

There are several methods that can achieve the ideal supersaturation state for nucleation and crystal growth. Most methods can be placed into one of three categories: vapor diffusion (VD), batch crystallization, or liquid-liquid diffusion [3, 14]. Vapor diffusion is the most extensively used method that includes two different techniques: hanging-drop vapor diffusion (HD-VD) or sitting-drop vapor diffusion (SD-VD). In both techniques, the protein and precipitant are equilibrated against the crystallization reservoir solution separated by an air gap. Their difference is simply as each name implies. In the SD-VD method, the protein/precipitant mixture resides in a well sitting above the reservoir solution. Whereas in the HD-VD method, the protein/precipitant mixture is hanging over the reservoir solution from an inverted glass slide. In each setting, water vapor diffuses from the drop into the reservoir solution slowly concentrating the protein/precipitant mixture, promoting supersaturation and ideally nucleation of the protein.

Batch crystallization is a method in which both concentrated protein and precipitant are mixed together and covered by a layer of paraffin oil [2, 3, 12, 15]. This technique can be used for very small volumes, often referred to microbatch crystallization with droplets as small as 1 µl [2, 16]. In batch crystallization, the crystallization conditions can be finely controlled due to the inability of air to penetrate the oil layer. Airborne contamination and other variables are blocked from contacting with the sample reducing interference in protein crystallization [2, 14]. Batch crystallization is also useful for producing large quantities of microcrystals suitable for serial crystallographic experiments [17].

Liquid-liquid diffusion (also known as counter diffusion) is a technique in which the protein and precipitant are injected on each side of a closed channel and gradually mixed through diffusion [3, 14, 18, 19]. At the beginning of mixing, the two solutions come into contact at their maximum concentrations in a reagent chamber, resulting in supersaturation and promoting spontaneous nucleation. As the mixing proceeds, the mixture reaches equilibrium and the level of supersaturation is decreased, consequently favoring crystal growth. This method can be performed in a variety of configurations; for example, microfluidic devices have been developed using capillaries and microchips which now allow for in situ X-ray data collection [14, 18].

## 3. Screening and Additives

In a typical crystallization experiment, thousands of conditions are often tested for a single protein in order to acquire a crystal suitable for X-ray diffraction. Variables that may affect crystallization include pH, temperature, and precipitant concentration. The pH is typically controlled by introducing a buffering agent into the crystallization condition. Buffering agents that are commonly used include Tris hydrochloride, HEPES, sodium cacodylate, MES, and sodium acetate. Precipitants are among the most variable factors and can be divided into four different categories based on their properties: salts, organic solvents, long chain polymers, and low-molecular-weight polymers and nonvolatile organic compounds [3]. Common salts include ammonium sulfate or sodium chloride whereas common organic solvents include ethanol and isopropanol. The polyethylene glycol family (PEG) such as PEG 3350 is representative of the third category whereas PEG 1000 or lower molecular weight PEG along with compounds such as methylpentanediol (MPD) are representative of the latter [3].

Additives can be classified as any foreign molecule introduced into the crystallization condition other than the aforementioned components. The purpose of adding additives is to facilitate or enhance crystal formation or growth. Examples may include small molecules, detergents, metal ions, or other various compounds. Additives do not necessarily promote supersaturation of the protein in solution but are intended to contribute to protein solubility or structural rigidity. These compounds can often perturb sample to sample and solvent to solvent interactions influencing the behavior of protein crystallization. There are several reports in which inclusion of additives resulted in improvement of both crystal size and quality [20, 21, 22]. However, screening numerous random molecules is tedious, and success is often limited. Thus, a more rational approach is the introduction of natural additives or compounds already found to interact with the protein of interest. These types of additives might include cofactors or ligands required for the biological activity of the protein. Such molecules not only facilitate successful crystallization but also provide functional insight into the protein by revealing the substrate or cofactor binding site [23, 24, 25].

## 4. Construct Optimization

Although supersaturation is the premise behind protein nucleation and crystallization, the protein itself can be a critical variable for the formation of a crystal and subsequent growth. It has been argued that the protein, rather than the crystallization condition, may be the most important variable in the crystallization process [26]. Solubility and monodispersion of the protein are often necessary in successful crystallization experiments. Non-specific aggregation by hydrophobic amino acids or flexible protein regions can interfere with directional nucleation and overall crystal lattice formation. Therefore, protein construct optimization is often implemented in protein crystallography. During the molecular biology boom of the 1980s and 1990s, proteins that had been previously understudied due to their low abundance in the cell could now be cloned, expressed, and purified in milligram quantities using bacterial expression systems [2, 3]. However, the technology of molecular cloning would not only pave the way for the study of previously unobtainable proteins, but also would allow for manipulation of protein constructs to facilitate X-ray crystallographic studies. Standard polymerase chain reaction (PCR) and recombinant DNA technology now allow for the deletion of protein regions that may interfere with crystallization. It is common practice in construct development for protein structural analysis to remove flexible amino acid sequences [26]. These regions can be identified by a variety of techniques such as limited proteolytic cleavage followed by fragment analysis, orthological structure comparison, and multiple sequence alignment [27, 28, 29]. Removal of the flexible regions can reduce conformational heterogeneity of the protein and enhance ordered formation of the crystal lattice. For example, deletion of the N- and C-terminal residues from *S. typhimurium* aspartate receptor ligand-binding domain has improved crystal diffraction from 3 to 1.85 Å [30, 31]. Deletion of the N-terminal residues and an internal flexible loop from *S. aureus* DNA gyrase has made crystals diffract from 3 Å to 2 Å [32].

## 5. Surface Residue Modification

Besides removal of problematic amino acid sequences from the protein, mutagenesis of surface residues may also be implemented to enhance the formation of crystal contacts. One of the first successful examples of this strategy was that of human ferritin by Lawson in 1991 in which some surface residues were mutated to promote the crystal contacts analogous to the structure of the rat isoform [33, 34]. Subsequent studies by other groups such as McElroy in 1992 with thymidylate synthase, Zhang in 1995 with T4 lysozyme, and Zhang in 1997 with leptin showed that mutagenesis of surface residues can greatly impact the formation of the crystal lattice [35, 36, 37].

Chemical modification of surface residues can also facilitate the formation of crystal contacts by reducing surface entropy of the protein. The most common approach has been the reductive methylation of primary amine groups by dimethylamine-borane in the presence of formaldehyde [38]. Residues subjected to such methylation include exposed lysine or arginine side chains and the N-terminal primary amine. This strategy offers some advantages over mutagenesis by eliminating the time-consuming process of protein production. Additionally, methylation is performed on the intact protein which prevents mutagenesis-induced improper folding of the nascent polypeptide [38, 39]. Furthermore, only residues exposed on the surface of the protein will be modified, and those buried in the core or residues responsible for strong protein-protein interfaces are not affected. However, this method of non-specific surface modification may eliminate residues critical for substrate binding or other biologically relevant interactions.

## 6. Fusion Tags for Protein Solubility

Unfortunately, even with direct construct optimization and surface modification of the target protein, crystallization success is no guarantee. Optimized constructs may still experience solubility or aggregation issues due to the improper folding of the target protein with bacterial expression systems. To circumvent these issues, molecular cloning strategies are often used to attach a solubility tag to the target protein to promote protein folding and stability. This is often accomplished by cloning the target protein into a vector that contains a protein tag which is known to fold well and exhibit substantial expression and solubility. The most common solubility tags used in crystallography experiments include Small Ubiquitin-like Modifier (SUMO), Glutathione S-transferase (GST), Thioredoxin (TRX), avidin/streptavidin tags, and Maltose Binding Protein (MBP) [28, 40–43]. Classically, once purified, these fusion tags are removed prior to crystallization using an engineered protease site in the linker region between the target protein and tag. In a sequential purification step, the tag and protease are separated from the target protein yielding the highly pure protein suitable for crystallization.

Incorporation of these large solubility tags has been shown to provide substantial benefits especially in bacterial expression systems. It has been estimated that nearly 50% of all overexpressed prokaryotic proteins have solubility issues using only a hexahistidine tag (His-tag) expression system [28, 44, 45]. Recombinant proteins of eukaryotic origin with a His-tag suffer even higher solubility issues [28, 46–49]. Approaches that are often explored to resolve such issues include altering expression conditions such as temperature and induction strategy, and exploring alternative bacterial expression strains or eukaryotic expression systems [28, 44]. However, the behavior of individual proteins can vary substantially, so that it is highly advantageous that a more universal strategy is implemented. Thus, the solubility tag approach has become widely adopted due to its noted success in protein structure elucidation [28].

## 7. Carrier Mediated Crystallography

As previously explained, large protein fusion tags are commonly used in structural biology for solubility enhancement and promoting proper folding of the target protein. It is common practice to remove these tags prior to crystal screening. This is because: (1) tagged proteins are less likely to form well-ordered diffracting crystals due to conformational heterogeneity resulting from the linker region; and (2) addition of a large fusion tag lends the possibility that the native structure of the target protein is changed or physiologically relevant interactions are altered. However, because the tags are often responsible for enhancing solubility and structural integrity, removal of them from the target protein can result in unwanted complications [28]. Common problems from tag removal include precipitation of the target protein and insufficient cleavage, both of which can result in reduced protein yield or poor quality of proteins. The alternative to such issues is to leave the protein tag attached for crystallization trials [28]. Although previously thought to be undesirable, this practice, known as carrier mediated crystallography, is now being used to facilitate crystallization of proteins that have proven difficult to crystallize including membrane proteins [50–55].

The concept of carrier mediated crystallography is that by leaving on the fusion tag, the tag not only promotes solubility of the target protein but also facilitates nucleation and crystal lattice formation by its mediated crystal contacts. This sequentially promotes the incorporation of the target protein into the crystal lattice which may not have been possible without the tag. An additional benefit of this technique is that the phase problem in X-ray crystallography can be easily solved by the molecular replacement method, since most commonly used fusion tags have previously solved structures. The structures of fusion tags also allow easy implementation of surface entropy reduction to further increase the chances of crystallization success. Among the successfully used fusion tags are MBP, GST, SUMO, and specific antibody fragments.

### 7.1. Maltose binding protein

The first protein structure reported using a fusion tag approach was reported by Center in 1998 in which two fragments from the ectodomain of human T cell leukemia virus type 1 (HTLV-1) were crystallized with Maltose Binding Protein (MBP) as the tag [50]. Since then, it was described by Waugh in 2016 that over 100 crystal structures using MBP as fusion tag have been solved [56]. MBP is a 42.5 kD *E. coli* protein responsible for the uptake of maltodextrin and promoting its catabolism. MBP exists as a monomer in solution and is divided into two distinct globular domains connected by three short polypeptide segments. The two globular domains are separated by a deep pocket that is responsible for the binding of its substrate maltose or other maltodextrins [57, 58]. MBP can undergo a significant conformational change upon binding to its substrates (Figure 1B). The substrate bound form displays a closed substrate binding pocket and is related to the substrate unbound form by a rigid motion of the two domains around the linking polypeptide hinge (Figure 1B). The two MBP conformations can give rise to different crystal contacts. Thus, crystallization of MBP fusion proteins is often screened with or without the addition of maltose in order to promote monodispersion of MBP’s bound and unbound conformations. This was shown to be critical in at least one structure, (PDB code: 3WAI), in which only the ligand free form could be crystallized [56, 59]. In other structures, different crystal forms were observed between the ligand bound and unbound forms [56, 60].

The MBP conformational state is not the only factor that affects MBP mediated crystallization. The linker region between MBP and the target protein is also an important variable. Eleven MBP fusion structures as noted by Waugh exhibit relatively long linkers designed for proteolytic cleavage [56]. This suggests that crystallization with the MBP tag in these cases was a fall back approach when crystallization attempts of the cleaved protein failed. There is also a prevalent consensus of short linkers including N, NS, HM, GS, GSS, AMD, GSSGSS, and NSSS [56]. The linker NSSS is one of the common linkers deposited in the Protein Data Bank and is characteristic of the expression vector pMAL-c2 [56, 61]. This linker is created by PCR amplification of the target protein sequence with introduction of an in-frame SacI restriction site at the N-terminus of the protein. However, the most common linker for MBP fusion constructs is that of NAAA which is the result of a three point mutations at the MBP C-terminus. These point mutations can be traced back to the crystal structure of HLTV-1 gp21 ectodomain fused to MBP [56, 62]. It was anticipated that gp21 would exist as a homotrimer and thus three charged residues near the C-terminus of MBP were changed to alanine to avoid electrostatic repulsion. These three alanine residues code for a NotI restriction site which allows for the in-frame ligation of the target protein into the vector [56]. The asparagine that proceeds the three alanine residues is a cloning artifact introduced from the pMAL-c2 vector. Although there is no definitive answer to whether this linker more likely facilitate MBP mediated crystallization, the predominance of solved structures with this linker sequence suggests a good starting point in pursuing MBP carrier crystallization.

To further the chances of obtaining a diffracting quality crystal, surface entropy reduction can be implemented together with the MBP carrier technique. In 2010, Moon describes a tandem fixed arm MBP/surface entropy reduction mutation system, in which both the linker region and MBP loop regions are optimized. Custom short linkers were designed to promote a “fixed arm” where the conformational flexibility of the linker would be minimized but not interfere with the structure of MBP or the protein itself [61]. Surface entropy reduction mutations were then introduced on the solvent exposed loop regions of MBP. Using this strategy, Moon was able to solve three previously unobtainable protein structures including 2-O-sulfotransferase (2OST) from *Gallus gallus*receptor for activated C-kinase 1 (RACK1) from *Arabidopsis thaliana*and Derp7 from *Dermatophagoides pteronyssinus*. Structural analysis showed that some of these surface entropy reduction mutations promoted crystal formation likely by reducing electrostatic repulsion in crystal contacts [61].

One major concern with the MBP mediated crystallization is potential interference of MBP with the native conformation of the target protein. Waugh in 2016 addressed this issue by investigation of 24 proteins with and without MBP as a fusion partner. Following analysis, it was found that the average r.m.s.d (root-mean-square-deviation) for the fused and non-fused structures averaged approximately 1 Å suggesting that MBP fusion caused little to no structural change [56]. Altogether, this suggests that the MBP mediated crystallization is a viable technique that can enhance the probability of crystallization success without influencing the native structure of the protein target.

### 7.2. Crystal structures with carriers other than MBP

Although the majority of crystal structures deposited in the PDB using the carrier approach utilize MBP as the fusion partner, a number of other structures exist using alternative protein tags. As described by Smyth in 2003, crystallization of GST fusion proteins have been reported for the DNA-binding domain of *Drosophila* DNA replication-related-element-binding factor and for mouse estrogen receptor hormone binding domain [28, 63, 64]. Carter and Schmidt in 2011 and 2012 revealed the crystal structures of the dynein motor domain of *Saccharomyces cerevisiae* with GST fusion [51, 52]. In addition to GST, yeast SUMO was also reported as a fusion partner in crystallization. The SUMO tag is widely implemented in protein purification because of its high solubility; however, it is typically cleaved off prior to crystallization [65, 66, 67]. Regardless, at least nine unique SUMO fusion protein structures have been deposited in the PDB including the C-terminal domain of Ebola virus VP30, thymidylate synthase, alpha-keto acid dehydrogenase phosphatase, and peptidyl-prolyl cis-trans isomerase. However, despite a limited amount of research on either GST or SUMO fusion, both tags may serve as a viable alternative for carrier mediated crystallography.

### 7.3. Antibodies as a carrier mediated approach

An alternative carrier approach to the fusion protein is the use of antibody fragments in the facilitation of crystal formation. In this technique, crystal contacts are mediated between antibodies specifically bound to the protein of interest. One of the first uses of this technique was for the crystallization of the HIV capsid protein p24 where traditional crystallization methods were unsuccessful [53, 54]. It was only after screening with antibody fragments that crystals suitable for X-ray diffraction were obtained. Antibodies can be divided into two regions that include the F_ab_ region and F_c_ region. The F_ab_ region contains the sites that can bind to antigens, whereas the F_c_ region allows for the generation of an immune response [68]. The F_ab_ region is composed of one variable domain and one constant domain from each the light chain and heavy chain of the antibody [69]. The variable domains are collectively known as the F_v_ region and is the most important region for binding antigens, constituting specificity and antigen discrimination.

When using antibodies as a carrier in crystallography, the most important part is selection and preparation of homogenous F_ab_ fragments specific for the protein target. Monoclonal antibodies are generated using a standard procedure and usually selected in an ELISA using the target protein as antigen [70]. The use of entire antibodies can hinder crystal lattice formation due to the flexibility between the F_ab_ and F_c_ [55, 71]. Thus, the use of F_ab_ fragments is the most common approach in this technique. One approach that can be used to isolate the F_ab_ fragments is to subject the antibodies to papain digestion which cleaves the flexible domain between the F_ab_ and F_c_ regions [53]. The resulting F_ab_ fragments are then purified by ion exchange chromatography to remove the F_c_ regions. Although the cleaved F_ab_ fragments are relatively easy to obtain, care must be taken in the purification procedure to ensure homogeneity of the fragments. The more definitive approach to generating identical F_ab_ or even F_v_ regions is the use of recombinant methods that can ensure homogenous fragments for crystallization [55].

Once purified, the fragments are then mixed with the respective protein target and standard crystallization procedures are implemented. As shown by Hunte and Michel in 2002, the crystal contacts in the KcsA K^+^ channel crystal are entirely mediated by the F_ab_ fragments, virtually suspending the target protein within the crystal lattice (Figure 1C) [55, 72]. The same was noted for the crystallization of cytochrome *c* oxidase (COX) where all crystal contacts are mediated by the bound F_v_ fragments (Figure 1C) [55, 73]. These observations suggest that the antibody fragments can provide substantial benefits in the mediation of protein crystallization. The high affinity of the antibody for its target eliminates the need for a linker as with the fusion method and reduces the conformational flexibility of the protein molecule. Additionally, as observed in the crystal structures of KscA and COX, crystal lattice formation can be achieved purely by the antibody fragments eliminating the need for the manipulation of crystal contacts from the target molecule. However, even with recombinant methodologies, this approach can be very costly and labor intensive making this strategy often one of last resorts in protein crystallography.

Nanobodies are a new addition to antibody assisted crystallography [74]. They are single-domain antibodies occurring naturally in Camelids. Like conventional antibodies, nanobodies are able to selectively bind to an antigen and have the full antigen-binding capacity with a molecular weight of only 12–15 kD. However, nanobodies exhibit the competitive advantage over conventional antibodies due to their superior stability and unique structural properties that allow access to the cavities or clefts of target proteins [74]. In protein crystallography, nanobodies have been applied to trap unstable structural intermediates, enhance rigidity of multidomain proteins, assist crystallization of intrinsically disordered proteins, and stabilize the protomers of large protein assemblies [75–79]. Because they usually recognize conformational epitopes, nanobodies are ideal tools to study the crystal structure of G protein coupled receptors (GPCR) and can stabilize the specific conformational states of the proteins [80, 81].

## 8. Nanotechnology in Protein Crystallography

Regardless of the method used to facilitate crystallization of a protein, crystal formation is limited by the laws of chemistry and the insurmountable variables involved in macromolecular interactions. When simplified, the three key components to crystallization success are nucleation, conformational stability, and ordered protein-protein contacts. With every strategy, there are strengths and limitations. However, technological advancement continues to open alternate pathways to overcome this barrier. Nanotechnology and the use of nanoparticles have been extensively explored in recent years due to its wide range of practical application in physics, optics, electronics, and even medicine [82, 83]. Nanoparticles can be defined as an ordered cluster of atoms, typically inorganic materials, that have at least one dimension between 1 and 10 nanometers. They tend to be highly reactive and have been used for conjugation to a variety of molecules with applications in protein crystallography.

An example of such technology was described by Ko in 2017 in which nanoparticles served as an inducing reagent in protein nucleation and crystal growth [82]. Ko demonstrated that formation of lysozyme nucleation cores can be accelerated by decoration of gold nanoparticles with -COOH and Ni^2+^ ions. The interactions between lysozyme and the immobilized -COOH and Ni^2+^ ions can readily conjugate lysozyme to the nanoparticles creating the nucleation core [82]. Manipulation of nanoparticle size and shape increased the number of successful crystallization conditions by 24%. Chen in 2017 described another approach for nucleation induction using nanodiamond (ND) carbon based particles. Like the gold particles described by Ko, NDs were modified with various oxygen containing groups including -COOH, -COH, and -C=O for protein conjugation [83]. Chen reported that the nanoparticles were able to increase the crystallization efficiency of several proteins including lysozyme, ribonuclease A, proteinase K, and catalase [83]. NDs were also able to effectively induce crystallization of lysozyme at concentrations as low as 5 mg/mL. This finding indicates that the nanoparticles can facilitate the crystallization of proteins at low concentration or at ultra-low supersaturation.

In addition to the nanoparticles, several other technological innovations are being explored as potential facilitators of crystallization. Crystallization mediated by porous materials such as silicon has been examined. Microfluidic devices have also been developed to increase protein crystallization efficiency [84, 85]. Crystallization of membrane proteins using lipidic bicelles and lipid cubic phase has been reported with variable success [86–89]. Racemic crystallography is another useful technique for facilitating crystallization. In this technique, the crystals are grown using a mixture of naturally occurring protein and its chemically synthesized mirror image [90]. Crystallization of this racemic mixture is easier than crystallization of a single enantiomer due to its ability to form favored centrosymmetric crystals [91]. Another advantage of this technique is the ease for structure determination. If sufficiently high resolution data could be measured, the phase problem can be solved by direct methods as the phase angles are restricted to 0° or 180° [92]. Since this technique was explored in 1993 using the small protein rubredoxin [93], the structures of numerous proteins have been solved by this method such as ShK toxin, ubiquitin, and plectasin [92, 94, 95]. Although no single technique has been successful for the crystallization of all proteins, each method can be implemented as a potential option for successful protein crystallization.

## 9. PDZ Domains in Nucleation and Crystal Facilitation

With efforts to expand the crystallization toolbox, our laboratory has recently begun to develop additional approaches to facilitate protein nucleation and crystal formation. One of our approaches was designed to simulate biological scaffolding process in which a protein-protein interaction known to mediate scaffolding of protein complexes was exploited as a carrier for crystallization. Because the essence of nucleation is ordered protein interaction which results in the formation of a crystal lattice, the use of scaffolding proteins as a carrier may increase the chance of well-ordered crystal contacts. The following will describe the potential of using the scaffolding properties of PDZ domains in facilitation of crystal lattice formation.

PDZ domains are composed of 80–90 amino acids that play critical roles in protein scaffolding and complex assembly at the cellular membrane. The acronym PDZ is derived from the first three proteins originally found to contain this domain. These three proteins are known as Postsynaptic density protein 95 kD (PSD95), Drosophila disc large tumor suppressor (Dig1), and Zonula occludens-1 protein (ZO-1) [96, 97]. PDZ domains bind to their targets by recognizing specific short C-terminal amino acid motifs of their targets. This structurally conserved interaction promotes scaffolding of protein complexes and is important for the assembly of signaling complexes, protein trafficking, and the recycling of cell receptors [98]. PDZ domains can be classified as either class I or class II based on their substrate specificity. Class I PDZ domains recognize the C-terminal peptide consensus sequence (S/T)X(V/I/L) (X denoting any amino acid) and class II recognizes (F/Y)X(F/V/A) [99, 100]. All PDZ domains share an evolutionary conserved fold consisting of six β-strands (β1–β6) and two α-helical segments (αA and αB). A similar peptide recognition mode is shared among all PDZ domains with the target peptide inserted between the strand β2 and helix αB [96, 99–104]. Many PDZ proteins increase their scaffolding capability through dimerization and promote formation of large macromolecular complexes [101, 105, 106, 107].

**Figure 2:**
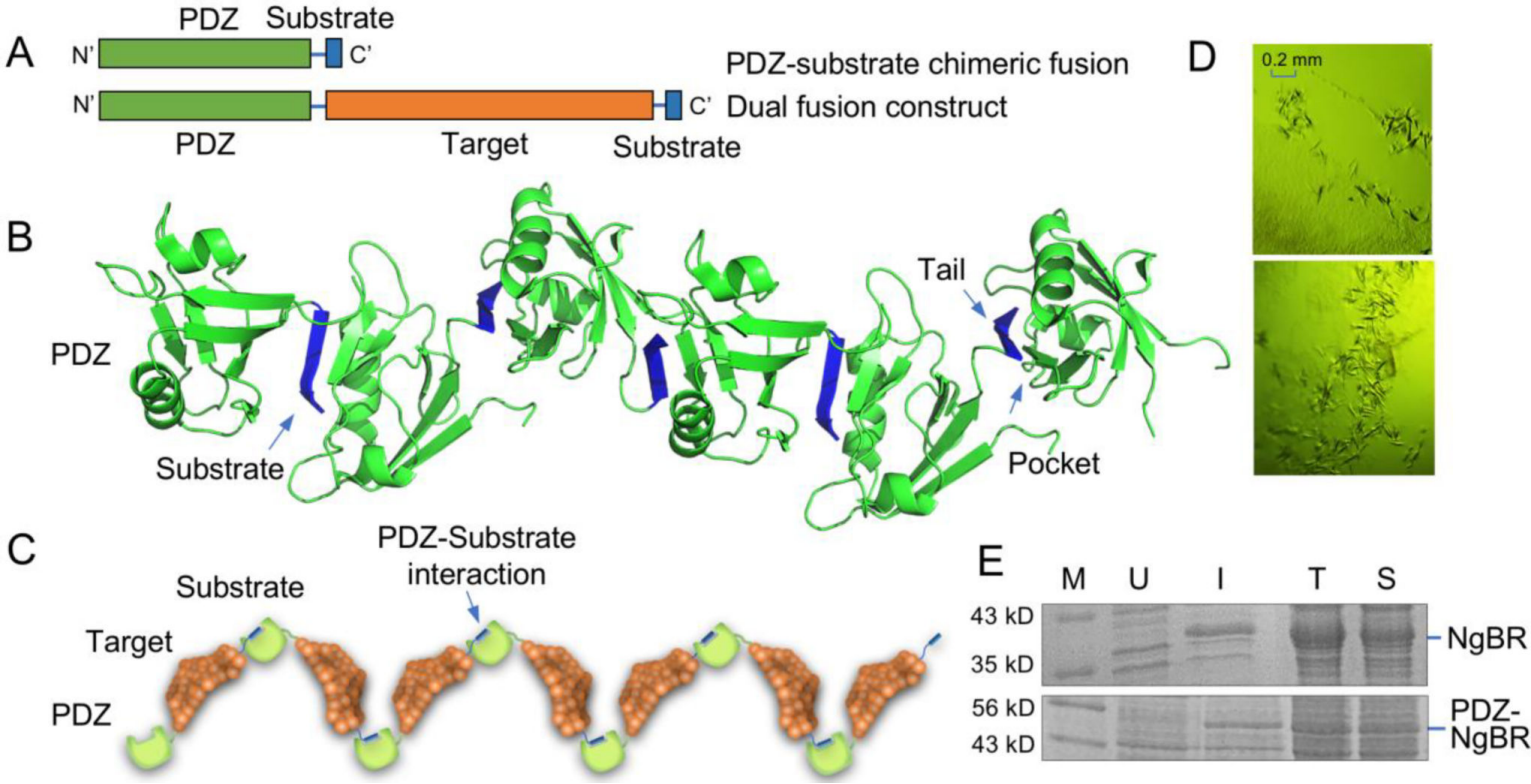
PDZ scaffold mediated protein crystallization. (A) A chimeric PDZ-substrate fusion construct (top) and a dual protein fusion construct with a target protein sandwiched by a PDZ domain and PDZ substrate peptide (bottom). The orange region denotes the target protein while the green region denotes the PDZ and blue is the PDZ substrate region. The same color scheme is used in Figure 2B and Figure 2C. (B) Crystal contacts mediated by the repeated “pocket and tail” interactions in the crystal of NHERF1 PDZ1-CXCR2 fusion protein (PDB code 4JL7). (C) Theoretical representation of crystal contact formation in PDZ scaffold mediated protein crystallization. (D) Crystals obtained for NHERF1/PDZ1-NgBR-CXCR2 (top) and NHERF1/PDZ1-SMYD5-CXCR2 (bottom). The size of the crystals is approximately 0.2 × 0.04 × 0.04 mm. NgBR, molecular weight of 24.4 kD and SMYD5, 47.3 kD. Proteins were expressed using pSUMO vector [23]. (E) Expression and solubility assessment of NgBR with and without N-terminal PDZ fusion. Note that both constructs contain a His-SUMO tag. Lane M, molecular weight marker; U, uninduced cell culture; I, induced cell culture; T, total cell lysate; S, supernatant of cell lysate.

Numerous PDZ-substrate complexes have been crystallized. One unique and effective strategy in these crystallizations is the use of a chimeric protein construct, i.e. the peptide ligand attached to the C-terminus of the PDZ molecule (Figure 2A) [101–104]. In the crystal, the chimeric protein displays a polymeric arrangement with the C-terminal ligand sequence bound to a neighboring PDZ, leading to the formation of a linear filament throughout the crystal (Figure 2B). This repeated “pocket and tail” interaction appears to facilitate directional nucleation contributing to crystal contact formation between adjacent PDZ molecules. This strategy has proven essential for the crystallization of NHERF1 (Na^+^/H^+^ Exchanger Regulatory Factor 1) PDZ1 which was not able to be crystallized without the peptide substrate fusion [104].

Inspired by this effective chimeric protein approach, we designed a dual fusion construct with the target protein sandwiched by a PDZ domain and its respective peptide ligand (Figure 2A). The goal of building this construct is to explore the potential of specific PDZ-ligand interaction in promoting the crystallization of other proteins. Since the crystallization of PDZ domains is facilitated by the attachment of the C-terminal peptide substrate, our concept is that adding a PDZ molecule to the N-terminus of the target protein and a specific PDZ substrate peptide to the protein C-terminus may facilitate nucleation (Figure 2C). The N-terminally fused PDZ domain would recognize the C-terminally fused peptide substrate from another fusion protein, creating a chain of interactions that would facilitate crystal lattice formation. As with antibody mediated crystallization, the target protein could be virtually suspended within the crystal lattice reducing the necessity for the formation of crystal contacts from the target protein itself. Although in its infancy, our laboratory has employed this strategy for two proteins, NgBR (Nogo-B receptor) and SMYD5 (SET and MYND domain containing protein 5), for which standard crystallization methods have been unsuccessful. Interestingly, small crystals were obtained for both proteins in very similar conditions suggesting that the crystallization was mediated by PDZ-ligand interaction (Figure 2D). In addition, PDZ domains are very soluble and stable in solution. Using them as a fusion partner could also increase the relative solubility of the target protein. This was verified in one instance when comparing the expression profile of NgBR with and without the N-terminally attached NHERF1 PDZ1 domain (Figure 2E). As shown in Figure 2E, the expression level was reduced with PDZ; however, when comparing lanes T and S, the relative solubility was increased from roughly 30% (without PDZ) to 60% (with PDZ). Thus, our dual fusion protein approach not only provides a molecular scaffold for crystallization but also is able to increase protein solubility. Optimization of this strategy may prove as an additional approach for crystallization of problematic proteins.

## 10. Conclusion Remarks

X-ray crystallography continues to be the leading method for the elucidation of protein structure and rational drug design. However, the unpredictability of protein crystallization can significantly suppress the rate at which such discoveries are made. Although no crystallization method has guaranteed success, numerous strategies have been employed in order to increase the probability of which it can occur. Adjustment of crystallization components, modification of the protein construct, addition of carrier molecules, or even synthetic materials can be used alone or in combination to increase the odds at which the target protein can be crystallized. Additionally, utilization of the natural PDZ scaffolding ability may be implicated as an additional strategy for the induction of nucleation as well as facilitating the formation of crystal contacts. Together, all the strategies reviewed here are viable approaches which may help evade the bottleneck of crystallography and advance the analysis of protein structures.
